# Integration of bovine herpesvirus 4 genome into cultured persistently infected host cell genome

**DOI:** 10.1186/1743-422X-7-246

**Published:** 2010-09-21

**Authors:** Gaetano Donofrio, Antonio Capocefalo, Valentina Franceschi, Lisa De Lorenzi, Vicky van Santen, Pietro Parma

**Affiliations:** 1 Dipartimento di Salute Animale, sezione di Malattie Infettive degli Animali, Università di Parma, Via del Taglio 10, 43100 Parma, Italy; 2 Dipartimento di Scienze Animali, Sezione di Zootecnica Agraria, Università di Milano, Via Caloria, 220133 Milano, Italy; 3 Department of Pathobiology, 264 Greene Hall, College of Veterinary Medicine, Auburn University, Auburn, Alabama 36849-5519, USA

## Abstract

Persistent infection of macrophages with bovine herpesvirus 4 (BoHV-4) has been proposed to play a secondary causal role, along with bacterial infection, in bovine post-partum metritis. Mechanisms of maintenance of BoHV-4 persistent infection are not understood. We previously generated in vitro models of BoHV-4 persistent infection in human rhadomyosarcoma and bovine macrophage cell lines by drug selection of cells infected with BoHV-4 carrying a drug-resistance marker, and demonstrated circular episomal BoHV-4 genomes. In the present study, we used fluorescent in situ hybridization (FISH) to demonstrate BoHV-4 genomes also integrated into the genomes of these persistently infected cells.

## Findings

Bovine herpesvirus 4 (BoHV-4), a member of the Gammaherpesvirinae subfamily, was first isolated in Europe from respiratory and ocular diseases by Bartha and colleagues [1] and later in the United States by Mohanty and colleagues [2]. BoHV-4 has been isolated from a variety of samples and cells from healthy cattle and from cattle with abortion, metritis, pneumonia, diarrhea, respiratory infection, and mammary pustular dermatitis [3]. However, only a few investigators have successfully produced experimental disease. Although no clear direct disease associations have been demonstrated, abundant evidence consistent with a secondary role for persistent infection by BoHV-4 in bovine post-partum metritis has accumulated [4]. Like other herpesviruses, BoHV-4 establishes persistent infections in its natural host [5,6] and in an experimental host, the rabbit [7]. Although BoHV-4 has been demonstrated in many tissues, accumulated evidence suggests that the main site of persistence in both natural and experimental hosts is cells of the monocyte/macrophage lineage [8]. Based on this and other evidence, a pathogenetic model of persistent BoHV-4 infection along with bacterial co-infection has been postulated. Bacterially induced metritis in cattle persistently infected with BoHV-4 could possibly be exacerbated or become chronic following the recruitment of macrophages persistently infected with BoHV-4 from the bloodstream to the site of inflammation [9,10]. This model could explain the fact that BoHV-4 can also be isolated from healthy animals, where, in the absence of inflammation, the pathogenic potential of BoHV-4 is ameliorated. Therefore, persistent infection represents a prerequisite for BoHV-4 potential pathogenicity. However little information is available about BoHV-4 persistent infection. We previously generated in vitro models of BoHV-4 persistent infection in a human rhabdomyosarcoma cell line, RD-4 [11], and a bovine macrophage cell line, BOMAC [12]. RD-4 cells and BOMAC cells were infected with the recombinant BoHV-4 26A3neo, which carries the neomycin-resistance gene, and infected cells were selected with geneticin (G418). In both cases, colonies developed from cells surviving both the virus infection and the drug selection. These colonies were cultivated into cell lines that could be passaged in the presence of the selective drug. These cells contained the BoHV-4 genome, as demonstrated both by in situ lysis gel analysis [also called Gardella gel electrophoresis, a method capable of distinguishing cellular genomic DNA from covalently closed circular DNA (episomes) and from linear viral DNA [13]] and by PCR. Therefore, the presence of the BoHV-4 genome was compatible with both the survival and replication of RD-4 and BOMAC cells. These cells also produced low amounts of infectious BoHV-4. The ability to select geneticin-resistant cells 30 passages after infection with recombinant BoHV-4 was further evidence that persistent infection could be established. Further, for BOMAC cells persistent infection could be established even in the absence of drug selection, as would be the case following the natural infection of cattle with wild-type virus. Although the persistence of the viral genome was well documented, its possible integration in the cellular host genome was not investigated.

The possibility of integration of the BoHV-4 genome into the genome of cells persistently infected with BoHV-4 was suggested by the following two observations: 1) When the physical state of BoHV-4 genome in the drug-selected BoHV-4 persistently infected cells was analysed by Gardella gel electrophoresis and Southern hybridization with a BoHV-4 probe, a specific signal besides episomal circular and linear BoHV-4 DNA, was detected at the loading well of the Gardella gel, corresponding to the host genomic fraction. That signal could correspond to integrated BoHV-4 genome. Alternatively, the signal associated with the host genomic fraction might be due to large, complex concatameric viral genome replication intermediates or to mishybridization of the probe with host genomic DNA. However, neither alternative seems to be the case, because such signal was absent from the genomic fraction of acutely infected control cells. 2) Using an alternative approach to determine the status of the BoHV-4 genome in persistently infected cells, based on Southern blot analysis of the complete cellular genome digested with a suitable restriction enzyme and hybridised with a specific viral DNA probe, multiple distinct fragments that specifically hybridised with probe were generated, instead of a single fragment of the expected size corresponding to episomal or linear non-integrated forms (unpublished results). This was not due to partial digestion, because control DNA from cells acutely infected with BoHV-4 generated a single fragment of the expected size. Therefore, size differences of the hybridising fragments could depend on different distances to restriction sites in linked cellular flanking sequences resulting from independent integration events.

Based on this evidence, the possibility of integration of the BoHV-4 genome into the host genome in persistently infected cells was further investigated. To provide independent verification of these observations, two independent BoHV-4 drug selected persistently infected cell lines, an RD-4 cell line [11] and a BOMAC cell line [12], passed for 30 passages and kept under G418 selection, were analysed by fluorescent in situ hybridization (FISH) with a probe corresponding to the entire BoHV-4 genome cloned as a bacterial artificial chromosome (BAC) [14,15]. This technique can readily demonstrate integrated BoHV-4 genomes in metaphase cells because of the identical labelling of the sister chromatids in the same chromosome. In contrast, cells containing episomes, which are always present in multiple copies, should demonstrate multiple single dots of hybridization signal, randomly associated with the chromosomes. Metaphase chromosome preparations were prepared from BoHV-4-persistently-infected RD-4 [11] and BOMAC [12] cells, after 30 passages in the presence of selection drug, at log phase of growth. Cells were blocked in metaphase with 1 μg/ml of colcemid (Sigma, Milano, Italy) in growth medium (Dulbecco's Modified Eagle Medium supplemented with 10% fetal bovine serum, 2 mM L-glutamine, 100 IU/ml penicillin and 10 μg/ml streptomycin) for 2 hours at 37°C in a humidified atmosphere of 95% air/5% CO_2_. Harvest of metaphase cells and chromosome spreads were done according to standard procedures [16]. Probe containing the full genome of BoHV-4 cloned as bacterial artificial chromosome and purified by standard method [14] was labelled with Cy3-dUTP (Amersham Bioscience, Milano, Italy) by nick translation (Roche, Milano, Italy). Species-specific BAC probes, the human BAC RP11-153M12 and the bovine BAC INRA-115C10, were labelled with biotin. Briefly, 500 ng of labeled probe were hybridized to chromosomes at 37°C in 2 × SSC, 50% (vol/vol) formamide, 10% (wt/vol) dextran sulfate containing 7 μg BLOCK-iT™ DNA (Boehringer, Mannheim, Germany) and 3 μg of sonicated salmon sperm DNA, in a volume of 25 μL. Posthybridization washing was at 60°C in 0.1× SSC. Cy3 was directly detected as a red signal, whereas biotin-labeled DNA was detected using FITC-conjugated avidin (green signal) (Vector laboratories, Burlingame, CA). The chromosomes were identified by simultaneous 4',6-diamidino-2-phenylindolo (DAPI) staining. Digital images were obtained using a Leica DMR epifluorescence microscope (Leica Imaging Systems Ltd., Cambridge, UK) equipped with a CCD camera (Cohu Inc., san Diego, CA). Cy3 and DAPI fluorescence signals, detected using specific filters, were recorded, pseudo-colored, and merged using QFISH software (Leica Imaging Systems Ltd., Cambridge, UK).

**Figure 1:**
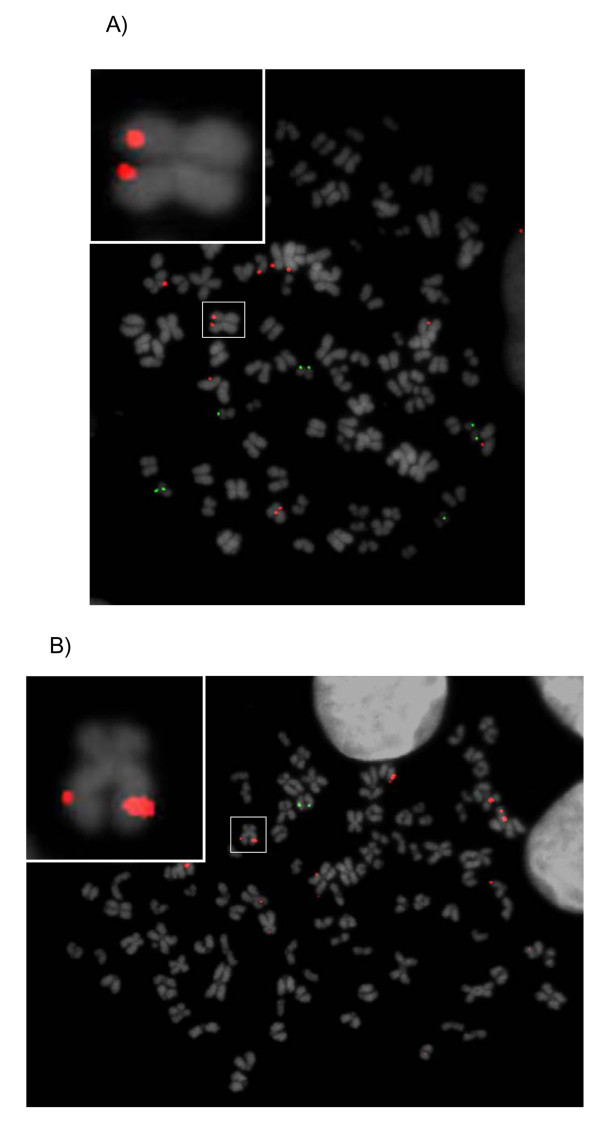
**Representative images of drug selected BoHV-4 persistently infected RD-4 **(A) **and BOMAC **(B) **cells, analyzed by FISH**. An example of BoHV-4 hybridization signals of symmetrically labelled sister chromatids in the same spread metaphase chromosome is indicated by a white square and enlarged as an insert picture at the corner of each image. Spread chromosomes were counterstained with DAPI. Red dot signals asymmetrically labelling the chromosomes correspond to episomally-maintained BoHV-4 genomes. Green signals show hybridization of species-specific BACs. The human BAC RP11-153M12 maps on HSA 20 at 20p13 and the bovine BAC INRA-115C10 maps on BTA 4 at a subcentromeric position.

Metaphase chromosomes from drug-selected BoHV-4 persistently infected RD-4 and BOMAC cell lines, showed specific fluorescent signals on their chromosomes, some of which showed symmetrical double signals on both chromatids (Fig. 1A and 1B). Observation of many chromatin spreads revealed integration at many different, apparently random sites. All of the metaphase spreads showed labelling and the hybridization efficiency and signal-to-noise ratio of this approach was very high. In parallel experiments, uninfected RD-4 and BOMAC or PHA-stimulated mononuclear cells from healthy bovines did not shown any BoHV-4 signals after hybridization (results not shown).

The molecular analyses described above suggest that, in the majority of BoHV-4 drug selected persistently infected RD-4 and BOMAC cells, the BoHV-4 genome can exist as a chromosomal integrated form. The previous observations made by Gardella gel electrophoresis and Southern analysis, suggesting the possible integration of the BoHV-4 genome, were thus corroborated. Although signals were present in identical positions on each sister chromatid, consistent with the presence of integrated DNA, other hybridization signals were also present (Fig. 1A and 1B), suggesting coexistence of integrated and episomal DNA. Further analysis would be required to unequivocally demonstrate such coexistence. Although this short report provides the direct visualization in vitro of the relationship between persistent BoHV-4 DNA with the cellular genome at the single cell level, many questions could be raised. First, does viral genome integration occur in vivo, or did the virus integrate in vitro during long-term cultivation? The second important question is concerned with the specificity of the cellular genomic site of BoHV-4 genome integration. Is integration random, or does it occur at specific chromosomal loci? Further work in different model systems, possibly in vivo using macrophage cells coming from persistently infected animals, will give an answer to the above questions. However, this is the first demonstration of the possible integration of BoHV-4 genome into the host genome.

## Competing interests

The authors declare that they have no competing interests.

## Authors' contributions

GD: Study design, performed the experiments, interpretation of the data and wrote the manuscript. AC: Contributed to perform the experiments. VF: Contributed to perform the experiments. LDL: Contributed to perform the experiments. VVS: Intellectually contributed. PP: Contributed to study design, performed the experiments and interpretation of the data. All authors read and approved the final manuscript.
